# Supplementary material for: A Likelihood Approach for Real-Time Calibration of Stochastic Compartmental Epidemic Models
Source: PLoS Comput Biol. 2017 Jan 17;13(1):e1005257. doi: 10.1371/journal.pcbi.1005257 (PMC5240920; doi:10.1371/journal.pcbi.1005257)
Supplement: S1 File — (TAR.GZ) [file pcbi.1005257.s014.tar.gz › HSPH_Online-SI-Revision/EnKF07/n1-extreme/n1-extreme_EnKF_table-8.pdf]

1	R0	{2.95019, 14.2854}	{1.79836, 8.82724}	{{0.469736, 2.00079}, {3., 17.18}}	{39.9149, 47.0766}	inf	3.	74.3276	{12.7822, 151.724}	2384.02	1.23051	newly	0.1	191.1	{8., 647.}	191 006.	2.7844	Reff	2.93839	1.45821	{0.328862, 2.6249}	50.4136	ar	924.	640.49	{143., 974.}	36.3236
2	R0	{2.67565, 16.946}	{2.60225, 7.07212}	{{2.13234, 2.50235}, {3., 13.0501}}	{15.8352, 58.2667}	inf	30.	214.321	{64.7524, 359.008}	616.608	0.758362	newly	15.	222.49	{62., 540.}	1384.33	1.01806	Reff	2.55525	0.824215	{0.230242, 1.6006}	67.7442	ar	926.	1558.79	{1087., 2118.}	71.351
3	R0	{2.40066, 16.6543}	{2.36914, 8.9853}	{{1.63407, 2.21331}, {3., 15.5786}}	{26.3937, 46.2569}	inf	14.	240.168	{70.064, 374.653}	1618.65	1.14062	newly	8.	272.32	{54., 689.}	3307.25	1.33509	Reff	2.34544	1.27886	{0.2434, 2.23993}	47.6972	ar	916.	1316.43	{803., 1838.}	50.4749
4	R0	{1.9294, 15.4361}	{2.23455, 6.9538}	{{0.965743, 2.33182}, {3., 15.8378}}	{46.8248, 59.3144}	inf	10.	264.413	{65.953, 502.1}	2544.13	1.31723	newly	5.	334.74	{58., 775.}	6594.8	1.64772	Reff	1.89467	1.04566	{0.37061, 1.8061}	47.1034	ar	796.	1338.7	{693., 1932.}	73.6181
5	R0	{2.92859, 19.5393}	{2.30009, 5.10774}	{{0.636468, 1.55818}, {3., 11.0907}}	{24.4846, 73.8591}	inf	28.	39.6832	{4.19994, 48.712}	102.933	0.386213	newly	16.	46.22	{3., 55.}	227.75	0.440322	Reff	2.7968	0.40767	{0.0994298, 0.688608}	85.4237	ar	927.	1747.	{1626., 1860.}	88.4574
6	R0	{1.79622, 12.3148}	{2.53944, 6.91939}	{{1.72636, 2.37137}, {3., 12.9841}}	{44.5351, 49.9942}	inf	35.	194.106	{75.3316, 336.152}	461.475	0.656116	newly	31.	190.49	{42., 466.}	521.516	0.674319	Reff	1.6651	0.659496	{0.192516, 1.06608}	64.2259	ar	734.	1560.81	{1081., 1979.}	113.029
7	R0	{2.45855, 17.1776}	{2.30015, 9.56004}	{{0.806112, 2.13956}, {3., 14.2068}}	{29.0066, 45.408}	inf	24.	192.727	{42.7371, 412.776}	707.695	0.796364	newly	13.	220.92	{21., 627.}	1604.31	1.01191	Reff	2.38971	1.44638	{0.20883, 2.75301}	44.6212	ar	871.	1135.51	{529., 2034.}	47.1814
8	R0	{2.20009, 15.519}	{2.02649, 5.27835}	{{0.225904, 1.25486}, {3., 7.202}}	{42.4048, 67.1392}	inf	26.	95.8866	{10.0305, 181.85}	289.869	0.481998	newly	18.	97.57	{9., 154.}	460.944	0.571567	Reff	2.00208	0.361366	{0.0452127, 0.530647}	82.2066	ar	839.	1641.45	{1483., 1812.}	98.6877
9	R0	{2.06493, 9.6414}	{1.83337, 9.29602}	{{0.420062, 2.00079}, {3., 18.2146}}	{43.6638, 51.3859}	inf	4.	74.8662	{18.8502, 152.843}	1776.18	1.12131	newly	3.	177.73	{11., 584.}	5831.	1.3688	Reff	2.04222	1.47009	{0.328514, 2.67043}	44.1362	ar	777.	677.05	{149., 1010.}	37.9163
10	R0	{2.60775, 19.6936}	{2.05868, 4.73528}	{{0.190903, 1.61761}, {3., 6.03539}}	{35.3852, 75.9552}	inf	29.	120.508	{11.9279, 366.415}	337.172	0.510351	newly	16.	129.08	{10., 330.}	719.	0.690714	Reff	2.47997	0.390462	{0.0357971, 0.614452}	84.2554	ar	889.	1638.59	{1236., 1988.}	90.3431
11	R0	{1.99168, 8.13555}	{2.30143, 9.43495}	{{0.925479, 2.14055}, {3., 14.1303}}	{37.9946, 44.6561}	inf	4.	199.476	{48.476, 428.116}	4888.38	1.56106	newly	3.	237.98	{32., 720.}	7834.	1.64712	Reff	1.97375	1.42289	{0.333766, 2.83206}	38.9158	ar	772.	1124.28	{572., 2038.}	57.7953
12	R0	{1.79304, 9.18941}	{1.79836, 8.82724}	{{0.469736, 2.00079}, {3., 17.18}}	{50.013, 53.8336}	inf	3.	74.3276	{12.7822, 151.724}	2384.02	1.23051	newly	4.	191.1	{8., 647.}	4686.5	1.30254	Reff	1.78587	1.45821	{0.328862, 2.6249}	45.0707	ar	725.	640.49	{143., 974.}	42.9807
13	R0	{1.84016, 18.3431}	{1.95883, 4.98907}	{{0.18519, 1.35359}, {3., 6.38645}}	{58.439, 72.8014}	inf	36.	108.209	{12.9532, 314.663}	228.315	0.433682	newly	12.	112.64	{10., 321.}	850.	0.729905	Reff	1.71319	0.36502	{0.0384887, 0.55335}	79.3105	ar	762.	1663.48	{1459., 2094.}	121.546
14	R0	{1.94221, 9.85971}	{2.33721, 6.75477}	{{0.972211, 2.33161}, {3., 15.108}}	{45.9532, 50.8996}	inf	16.	289.413	{79.0785, 541.227}	1710.35	1.15725	newly	13.	359.86	{68., 777.}	2670.92	1.27587	Reff	1.86646	1.07657	{0.384775, 1.79882}	46.0687	ar	805.	1365.97	{727., 1959.}	76.0882
15	R0	{1.78578, 12.0541}	{2.55257, 5.47331}	{{2.1259, 0.539398}, {3., 9.7562}}	{47.6051, 55.7914}	inf	16.	60.1656	{14.3459, 122.662}	285.931	0.454421	newly	14.	106.83	{14., 239.}	672.643	0.546481	Reff	1.68578	0.60006	{0.127901, 1.55363}	69.0914	ar	732.	1837.68	{1620., 2445.}	151.959
16	R0	{1.94463, 15.6503}	{2.29736, 9.28695}	{{0.842806, 2.13956}, {3., 13.7208}}	{39.9686, 42.9365}	inf	8.	196.948	{48.3416, 412.455}	2363.8	1.2672	newly	4.	246.31	{31., 732.}	6059.75	1.54042	Reff	1.92518	1.44011	{0.334568, 2.83108}	38.0025	ar	757.	1112.48	{547., 1982.}	60.0528
17	R0	{2.08901, 16.4314}	{2.61547, 7.0074}	{{2.15876, 2.50079}, {3., 12.9786}}	{28.655, 57.3902}	inf	17.	207.855	{64.4614, 358.229}	1123.96	0.998093	newly	8.	227.85	{60., 575.}	2748.13	1.29296	Reff	2.03887	0.862462	{0.247612, 1.60309}	58.9788	ar	826.	1569.42	{1095., 2173.}	92.155
18	R0	{2.76419, 18.56}	{2.28753, 9.4839}	{{0.811482, 2.14104}, {3., 14.2212}}	{25.5028, 49.1164}	inf	14.	190.367	{43.3521, 413.735}	1262.36	1.0026	newly	7.	224.49	{18., 639.}	3109.29	1.25569	Reff	2.70338	1.40455	{0.201691, 2.75849}	50.013	ar	920.	1115.69	{533., 1892.}	40.875
19	R0	{2.3821, 17.1399}	{1.7744, 9.03401}	{{0.375739, 2.00079}, {3., 18.2892}}	{41.8194, 49.9485}	inf	12.	73.8595	{15.0041, 151.295}	524.873	0.693955	newly	10.	179.39	{10., 579.}	1706.9	0.94841	Reff	2.34637	1.44492	{0.327821, 2.68326}	44.5757	ar	883.	644.49	{135., 998.}	36.5379
20	R0	{2.78314, 19.6127}	{2.10063, 5.09019}	{{0.231828, 1.43134}, {3., 7.35788}}	{30.9305, 74.0793}	inf	70.	107.791	{13.342, 169.868}	88.2859	0.266894	newly	32.	119.29	{18., 207.}	293.594	0.444085	Reff	2.44916	0.415594	{0.0414893, 0.62425}	83.3221	ar	943.	1583.19	{675., 1796.}	76.9618
21	R0	{1.85124, 14.6794}	{2.60254, 7.0808}	{{2.13089, 2.5084}, {3., 12.9837}}	{43.7711, 51.9931}	inf	18.	220.723	{66.7481, 376.782}	1129.75	0.999128	newly	15.	228.28	{60., 546.}	1425.47	1.04941	Reff	1.78274	0.853825	{0.236484, 1.60419}	56.3152	ar	730.	1566.69	{1091., 2179.}	116.281
22	R0	{2.1028, 14.9852}	{2.24458, 6.7177}	{{0.944906, 2.33081}, {3., 15.7618}}	{41.1155, 60.2703}	inf	26.	269.935	{65.5111, 511.728}	938.21	0.916532	newly	17.	348.53	{60., 796.}	1953.	1.1509	Reff	2.01659	1.02309	{0.354378, 1.80412}	50.7139	ar	840.	1335.33	{729., 1886.}	65.9702
23	R0	{1.86601, 10.2157}	{2.29757, 9.28274}	{{0.842517, 2.14005}, {3., 13.6895}}	{43.6354, 34.6637}	inf	8.	196.936	{48.3258, 412.688}	2363.57	1.26377	newly	6.	244.95	{28., 734.}	3984.17	1.35859	Reff	1.84548	1.43881	{0.334111, 2.83078}	38.4976	ar	747.	1115.31	{545., 1991.}	61.498
24	R0	{2.34032, 10.0992}	{1.79836, 8.82724}	{{0.469736, 2.00079}, {3., 17.18}}	{41.132, 47.8094}	inf	2.	74.3276	{12.7822, 151.724}	3621.04	1.389	newly	1.	191.1	{8., 647.}	19 016.	1.8444	Reff	2.3333	1.45821	{0.328862, 2.6249}	43.4655	ar	887.	640.49	{143., 974.}	36.4724
25	R0	{1.91548, 15.3401}	{2.26806, 6.78887}	{{0.946657, 2.32346}, {3., 15.8374}}	{48.8713, 60.3059}	inf	10.	269.63	{64.9917, 513.62}	2596.3	1.33096	newly	8.	345.32	{57., 783.}	4216.75	1.45892	Reff	1.88292	1.04161	{0.391303, 1.73564}	47.0635	ar	765.	1341.67	{697., 1892.}	80.3203
26	R0	{2.75283, 10.6721}	{2.30098, 9.44314}	{{0.922814, 2.13956}, {3., 14.1951}}	{25.1697, 33.4125}	inf	2.	199.509	{48.5023, 428.739}	9877.08	1.85961	newly	1.	237.26	{27., 724.}	23 628.	2.12244	Reff	2.73907	1.42572	{0.334728, 2.83267}	49.6955	ar	926.	1123.1	{525., 2038.}	40.9741
27	R0	{2.08367, 19.7849}	{2.21169, 7.32393}	{{1.21318, 4.42447}, {3., 14.642}}	{37.1896, 62.9822}	inf	51.	139.307	{20.212, 263.09}	197.727	0.42404	newly	27.	120.64	{14., 293.}	363.111	0.52723	Reff	1.89822	0.517573	{0.141799, 1.26171}	74.3644	ar	833.	1604.15	{1166., 2090.}	94.928
28	R0	{2.64334, 17.1765}	{2.32691, 9.76361}	{{1.29833, 2.29359}, {3., 15.8931}}	{24.9471, 43.7639}	inf	29.	246.27	{66.5717, 399.127}	756.341	0.855142	newly	16.	243.88	{49., 629.}	1432.38	1.03773	Reff	2.5191	1.32093	{0.23522, 2.23393}	48.3486	ar	934.	1321.89	{724., 1865.}	49.3587
29	R0	{2.87008, 13.3872}	{2.52572, 7.31867}	{{1.62687, 2.51528}, {3., 12.8143}}	{16.7061, 50.1552}	inf	6.	196.654	{61.1796, 337.291}	3180.06	1.36321	newly	4.	185.85	{36., 399.}	4548.25	1.48043	Reff	2.82129	0.685361	{0.260258, 1.06448}	75.7075	ar	941.	1566.2	{1180., 1962.}	68.9883
30	R0	{2.06105, 19.6275}	{2.25561, 5.41137}	{{0.62757, 1.26401}, {3., 12.703}}	{47.9826, 72.4296}	inf	103.	75.7194	{6.12884, 213.59}	99.7565	0.666911	newly	49.	84.05	{7., 184.}	153.98	0.475305	Reff	1.55609	0.407121	{0.103065, 0.659409}	75.1209	ar	747.	1823.66	{1638., 2203.}	144.131
31	R0	{2.30764, 13.435}	{2.30098, 9.44314}	{{0.922814, 2.13956}, {3., 14.1951}}	{31.0133, 35.8979}	inf	14.	199.509	{48.5023, 428.739}	1327.71	1.03513	newly	11.	237.26	{27., 724.}	2060.18	1.10888	Reff	2.26841	1.42572	{0.334728, 2.83267}	43.8228	ar	877.	1123.1	{525., 2038.}	45.0285
32	R0	{2.30565, 11.9237}	{2.27618, 9.29456}	{{0.82346, 2.14005}, {3., 14.1858}}	{31.5591, 34.0334}	inf	8.	190.73	{37.4399, 413.491}	2285.79	1.22609	newly	11.	227.12	{24., 638.}	1968.18	1.08732	Reff	2.26876	1.40302	{0.202327, 2.04574}	43.5429	ar	853.	1119.28	{499., 1902.}	48.83
33	R0	{2.24031, 18.582}	{2.53116, 7.41924}	{{1.62644, 2.49987}, {3., 12.8751}}	{25.2043, 60.5061}	inf	23.	198.86	{65.054, 333.81}	769.126	0.827683	newly	10.	179.26	{44., 365.}	1695.6	1.10646	Reff	2.17982	0.67929	{0.277105, 1.02296}	69.7612	ar	864.	1581.84	{1194., 2205.}	84.7963
34	R0	{2.33306, 15.9759}	{2.31141, 9.32324}	{{1.45996, 2.2081}, {3., 15.6535}}	{27.4852, 43.0054}	inf	16.	253.029	{68.3394, 559.919}	1484.84	1.09134	newly	12.	269.75	{49., 701.}	2153.42	1.16351	Reff	2.27473	1.29075	{0.234407, 2.23906}	45.9998	ar	854.	1349.98	{675., 1863.}	65.1288
35	R0	{2.48357, 18.3573}	{2.27407, 9.25355}	{{0.841215, 2.13956}, {3., 13.7303}}	{28.2412, 49.9407}	inf	17.	191.631	{51.8015, 412.294}	1030.2	0.952927	newly	9.	245.87	{32., 724.}	2634.78	1.20372	Reff	2.43887	1.43915	{0.191447, 2.4922}	45.2841	ar	835.	1112.87	{508., 1988.}	50.279
36	R0	{2.59548, 19.9046}	{2.59473, 7.05452}	{{2.16136, 2.52089}, {3., 13.0454}}	{17.173, 64.5583}	inf	18.	214.417	{66.7481, 372.257}	1092.45	0.981828	newly	13.	227.81	{62., 525.}	1653.15	1.0913	Reff	2.50983	0.855937	{0.233975, 1.61265}	65.8966	ar	863.	1574.56	{1092., 2202.}	84.8992
37	R0	{2.29641, 16.9868}	{2.42758, 6.44442}	{{1.61796, 4.17652}, {3., 8.73211}}	{28.3171, 62.0622}	inf	21.	63.3905	{8.12097, 119.126}	226.101	0.327894	newly	10.	72.11	{6., 131.}	636.1	0.530053	Reff	2.18159	0.535383	{0.153572, 0.805172}	75.8009	ar	877.	1836.54	{1647., 2260.}	110.363
38	R0	{2.65743, 15.4569}	{2.60307, 7.10678}	{{2.13547, 2.50548}, {3., 12.975}}	{15.9678, 54.1793}	inf	29.	213.623	{64.5515, 358.677}	638.877	0.771477	newly	16.	217.22	{59., 465.}	1259.13	0.987292	Reff	2.52987	0.822511	{0.230294, 1.59517}	67.4881	ar	897.	1557.85	{1075., 2130.}	76.5385
39	R0	{2.67953, 14.5132}	{2.27407, 9.25355}	{{0.841215, 2.13956}, {3., 13.7303}}	{26.3792, 39.9597}	inf	2.	191.631	{51.8015, 412.294}	9483.51	1.85805	newly	1.	245.87	{32., 724.}	24 489.	2.13377	Reff	2.67149	1.43915	{0.191447, 2.4922}	48.2671	ar	894.	1112.87	{508., 1988.}	44.104
40	R0	{1.94594, 11.928}	{2.28468, 7.05198}	{{0.950101, 2.33068}, {3., 15.7614}}	{45.9041, 54.6113}	inf	31.	267.561	{65.1642, 526.099}	763.099	0.827764	newly	31.	327.71	{52., 756.}	961.258	0.86786	Reff	1.84475	1.02551	{0.328195, 1.79126}	47.7279	ar	796.	1342.22	{660., 1889.}	75.206
41	R0	{1.66297, 10.0047}	{2.58384, 5.15283}	{{2.13205, 0.402465}, {3., 7.12259}}	{57.6265, 49.5754}	inf	25.	48.5796	{7.20121, 97.9653}	123.516	0.347378	newly	18.	88.01	{5., 159.}	413.611	0.491571	Reff	1.51995	0.581041	{0.132687, 1.53087}	69.0246	ar	716.	1774.11	{1608., 2292.}	149.823
42	R0	{2.91568, 16.4453}	{1.79836, 8.82724}	{{0.469736, 2.00079}, {3., 17.18}}	{39.861, 49.738}	inf	9.	74.3276	{12.7822, 151.724}	735.573	0.805502	newly	8.	191.1	{8., 647.}	2300.5	1.04422	Reff	2.88652	1.45821	{0.328862, 2.6249}	49.5585	ar	919.	640.49	{143., 974.}	36.3145
43	R0	{2.55898, 15.8889}	{2.29736, 9.28695}	{{0.842806, 2.13956}, {3., 13.7208}}	{27.5851, 43.5831}	inf	3.	196.948	{48.3416, 412.455}	6466.79	1.68465	newly	3.	246.31	{31., 732.}	8112.33	1.66286	Reff	2.54619	1.44011	{0.334568, 2.83108}	46.9861	ar	904.	1112.48	{547., 1982.}	43.354
44	R0	{2.28649, 15.9017}	{2.24061, 6.73742}	{{0.943923, 2.31784}, {3., 15.803}}	{35.4001, 61.2609}	inf	13.	269.787	{64.547, 511.771}	1975.28	1.21879	newly	10.	344.3	{56., 767.}	3343.8	1.36366	Reff	2.24076	1.01797	{0.36652, 1.82113}	54.755	ar	880.	1329.54	{683., 1884.}	59.2227
45	R0	{2.65121, 18.6339}	{1.77844, 9.04943}	{{0.416104, 2.00079}, {3., 17.6994}}	{40.6307, 52.1058}	inf	4.	75.5783	{14.8175, 151.91}	1796.32	1.12695	newly	1.	184.41	{10., 594.}	18 347.	1.82079	Reff	2.6353	1.43745	{0.329157, 2.68647}	46.93	ar	928.	629.63	{136., 984.}	37.4731
46	R0	{2.52291, 18.0251}	{2.26551, 6.87097}	{{0.962241, 2.32579}, {3., 15.794}}	{30.2473, 62.9231}	inf	13.	263.882	{65.7953, 502.281}	1929.86	1.20174	newly	3.	333.73	{54., 767.}	11 024.3	1.8666	Reff	2.4775	1.0375	{0.366899, 1.82035}	58.1229	ar	894.	1315.	{696., 1932.}	57.7293
47	R0	{2.22618, 17.1225}	{2.358, 9.20279}	{{1.62951, 2.21275}, {3., 15.5886}}	{29.4253, 46.4545}	inf	9.	238.553	{68.9857, 374.474}	2553.19	1.32248	newly	3.	256.98	{54., 626.}	8468.	1.73135	Reff	2.18388	1.25996	{0.240037, 2.20253}	46.0332	ar	850.	1321.5	{819., 1851.}	61.1459
48	R0	{2.04405, 16.073}	{2.38602, 9.92436}	{{1.13233, 4.67624}, {3., 15.9751}}	{36.9129, 39.8887}	inf	18.	262.002	{67.2729, 586.686}	1359.63	1.04751	newly	12.	247.32	{52., 604.}	1967.83	1.14635	Reff	1.96229	1.32847	{0.231178, 2.24013}	39.1781	ar	779.	1360.48	{763., 1884.}	78.2054
49	R0	{2.84396, 18.097}	{1.77844, 9.04943}	{{0.416104, 2.00079}, {3., 17.6994}}	{40.5685, 51.2062}	inf	14.	75.5783	{14.8175, 151.91}	451.186	0.649998	newly	11.	184.41	{10., 594.}	1590.82	0.91682	Reff	2.79846	1.43745	{0.329157, 2.68647}	48.9726	ar	952.	629.63	{136., 984.}	38.104
50	R0	{1.85341, 18.0975}	{2.37508, 9.93189}	{{1.34897, 5.87228}, {3., 15.4833}}	{42.5973, 45.2527}	inf	61.	242.008	{25.8452, 467.733}	312.105	0.561955	newly	26.	214.74	{17., 353.}	739.154	0.798153	Reff	1.67178	1.28176	{0.194022, 1.89223}	33.838	ar	754.	1316.99	{676., 1881.}	81.0968
